# Ovarian cancer subtypes based on the regulatory genes of RNA modifications: Novel prediction model of prognosis

**DOI:** 10.3389/fendo.2022.972341

**Published:** 2022-12-05

**Authors:** Peixian Zheng, Na Li, Xianquan Zhan

**Affiliations:** ^1^ Shandong Key Laboratory of Radiation Oncology, Shandong Cancer Hospital and Institute, Shandong First Medical University, Jinan, Shandong, China; ^2^ Medical Science and Technology Innovation Center, Shandong First Medical University, Jinan, Shandong, China

**Keywords:** ovarian cancer, RNA-modification regulatory gene (RRG), differentially expressed RRG (DERRG), RNA modification-related model, tumor immune microenvironment, risk score

## Abstract

**Background:**

Ovarian cancer (OC) is a female reproductive system tumor. RNA modifications play key roles in gene expression regulation. The growing evidence demonstrates that RNA methylation is critical for various biological functions, and that its dysregulation is related to the progression of cancer in human.

**Method:**

OC samples were classified into different subtypes (Clusters 1 and 2) based on various RNA-modification regulatory genes (RRGs) in the process of RNA modifications (m1A, m6A, m6Am, m5C, m7G, ac4C, m3C, and Ψ) by nonnegative matrix factorization method (NMF). Based on differently expressed RRGs (DERRGs) between clusters, a pathologically specific RNA-modification regulatory gene signature was constructed with Lasso regression. Kaplan-Meier analysis and receiver operating characteristic (ROC) curves were used to evaluate the prognostic ability of the identified model. The correlations of clinicopathological features, immune subtypes, immune scores, immune cells, and tumor mutation burden (TMB) were also estimated between different NMF clusters and riskscore groups.

**Results:**

In this study, 59 RRGs in the process of RNA modifications (m1A, m6A, m6Am, m5C, m7G, ac4C, m3C, and Ψ) were obtained from TCGA database. These RRGs were interactional, and sample clusters based on these regulators were significantly correlated with survival rate, clinical characteristics (involving survival status and pathologic stage), drug sensibility, and immune microenvironment. Furthermore, Lasso regression based on these 21 DERRGs between clusters 1 and 2 constructed a four-DERRG signature (ALYREF, ZC3H13, WTAP, and METTL1). Based on this signature, 307 OC patients were classified into high- and low-risk groups based on median value of riskscores from lasso regression. This identified signature was significantly associated with overall survival, radiation therapy, age, clinical stage, cancer status, and immune cells (involving CD4+ memory resting T cells, plasma cells, and Macrophages M1) of ovarian cancer patients. Further, GSEA revealed that multiple biological behaviors were significantly enriched in different groups.

**Conclusions:**

OC patients were classified into two subtypes per these RRGs. This study identified four-DERRG signature (ALYREF, ZC3H13, WTAP, and METTL1) in OC, which was an independent prognostic model for patient stratification, prognostic evaluation, and prediction of response to immunotherapy in ovarian cancer by classifying OC patients into high- and low-risk groups.

## Introduction

Ovarian cancer (OC) is a malignant gynecological disease in female reproductive system, which evolves as the eighth most frequent type of cancer and also the eighth most conventional death cause in women, accounting for 3.4% and 4.4%, respectively (1). OCs could be classified into epithelial and non-epithelial subtypes. According to histological characteristics of tumor cells, epithelial ovarian cancers are classified into mucinous, serous, endometrioid, or clear cell (2, 3). Different subtypes of OCs significantly influence its prognosis and should be treated in diverse therapy strategies (4). It is reported that 5-year relative survival is below 45% (5),which means there is a poor prognosis in OCs. Currently, surgical cytoreduction remains the main treatment of OCs, followed by neoadjuvant chemotherapy; targeted treatments such as poly ADP-ribose polymerase inhibitors and bevacizumab gradually become the maintenance treatment in the first line of clinical practice (6). Immunotherapy in OCs is getting increasing attention, and the predictiveness of response to immunotherapy may be improved by evaluating sensitive and resistant targeted therapy subpopulations on the basis of tumor biomarkers (7).

RNA modification is an addition of a chemical group on RNA nucleotide chains to regulate the functions of RNA biological behaviors with reference to post-transcriptional regulation (8, 9), which is also called epitranscriptome (10). Up to 170 types of chemical modifications have been discovered in RNAs (11), among which N1-methyladenosine (m1A), N6-methyladenosine (m6A), 2-O-dimethyladenosine (m6Am), 5-methylcytosine (m5C), N7-methyladenosine (m7G), N4-acetylcytidine (ac4C), 3-methylcytidine (m3C), and pseudouridine (Ψ) were especially critical. The process of RNA modification was regulated by three distinct clusters of specific proteins called writers, readers, and erasers (9, 12). Writers catalyze the formation of a specific chemical modification on RNAs; erasers catalyze the elimination of a specific chemical modification from the modified RNAs; and readers are RNA-binding proteins that could recognize and bind the modified RNAs (10). Previous studies identified different varieties of writers, erasers and readers of m1A, m6A, m6Am, m5C, m7G, ac4C, m3C, and Ψ. For m6A, its writer genes have KIAA1429, ZC3H13, METTL3, METTL14, WTAP, RBM15, and RBM15B, which catalyzed m6A methylation; Erasers included FTO and ALKBH5, which could reverse m6A modification through demethylation change; and readers contained YTHDC1, YTHDC2, YTHDF1, YTHDF2, HNRNPC, IGF2BP1, IGF2BP2, YTHDF3, IGF2BP3, HNRNPA2B1, and RBMX, which could recognize and bind the modified RNAs (13, 14). For m5C, writers (m5C methyltransferases) included TRDMT1, DNMT1, DNMT3A, DNMT3B, NSUN1, NSUN2, NSUN3, NSUN4, NSUN5, NSUN6, and NSUN7; erasers encompassed TET1, TET2, and TET3; and readers included YBX1 and ALYREF (15). For m1A, writers involved TRMT6, TRMT61A, TRMT61B, TRMT10C, and RRP8; erasers included ALKBH1 and ALKBH3; and readers included YTHDF1, YTHDF2, YTHDF3, and YTHDC1 (9, 16, 17). For ac4C, writers included NAT10 and THUMPD1; and erasers and readers remain unknown (18, 19). For m3C, only one writer METTL8 was discovered, and erasers and readers were still undetected (20). For m6Am, writers included PCIF1, METTL3, and METTL4; eraser involved FTO; and no readers were discovered (21–24). For m7G, writers included RNMT, METTL1, and WDR4; the only detected eraser was NUDT16, and readers were still unknown (25, 26). For Ψ, only writers were known, including PUS1, PUS3, PUS4, PUS7, PUS9, TRUB1, and TRUB2 (9, 10).

With the gradual in-depth studies of RNA modifications, an increasing number of RNA-modification regulatory genes (RRGs) were proved to play crucial roles in the occurrence and development of OCs. For instance, ALKBH3 affected the prognosis of OCs through inducing m1A demethylation to increase the CSF-1 stability (27). m6A demethylase ALKBH5 accelerated the process of ovarian carcinogenesis through NF-κB pathway in a simulated tumor microenvironment (28). DNMT3A/3B interacted with microRNA-29b in a double-negative feedback way to result in OC progression (29). FTO played a role in the acceleration of cell proliferation, inhibition of apoptosis, and autophagy activation in OC cells (30). The upregulation of HNRNPA2B1 in OC tissue promotes the proliferation of OC cells, which suggests that the upregulation of HNRNPA2B1 was associated with poor prognosis of OCs (31). IGF2BP1 enhanced the invasion of OC cells through inhibiting miRNA impairment gene expression (32). Elevated levels of IGF2BP3 and RNA-binding protein Lin28B were related to platinum chemoresistance as well as poor prognosis of OCs (33). From these studies, it is obvious that different varieties of RRGs significantly influenced the tumorigenesis, tumor progression, tumor aggressiveness, tumor cell proliferation, and drug resistance.

To search for novel cancer treatment strategies, tumor immune microenvironment (TIM) and immunotherapy became a new research hotspot. A study found that EZH2-mediated H3K27me3 along with DNMT1-mediated DNA methylation inhibited generation of Th1 chemokines, including CXCL9 and CXCL10, which helped effector T cells migrate to microenvironment in tumor. In that study, epigenetic modulators were used to eliminate the inhibition in tumor-bearing mice, increasing tumor infiltration of T cells, retarding progression of tumor, and promoting response to PD-L1 checkpoint blockade along with adoptive T cell transfusion. Further, EZH2 in combination with DNMT1 had a negative correlation with CD8+ T cells tumor infiltration and prognosis of OC patients (34). Another study certified the strong physical relationship between RNA-binding ubiquitin ligase MEX3A and IGF2BP2, PABPC1, LAMTOR2, and KHDRBS2, indicating the intense correlation of MEX3A expression level and infiltration of neutrophils, macrophages, dendritic cells, B cells, and CD8+ T cells. Activation of immune cells and immune modulators was related to decrease of mortality rate in OC patients. Additionally, the relevance of MEX3A and lymphocytes (neutrophils, macrophages, dendritic cells, B cells, and CD8+ T cells), immune stimulators, immune inhibitors, and MHC molecules was detected (35). Thereby, RRGs could affect TIM and play crucial roles in prediction of immunotherapy outcomes of OC treatment.

**Figure 1:**
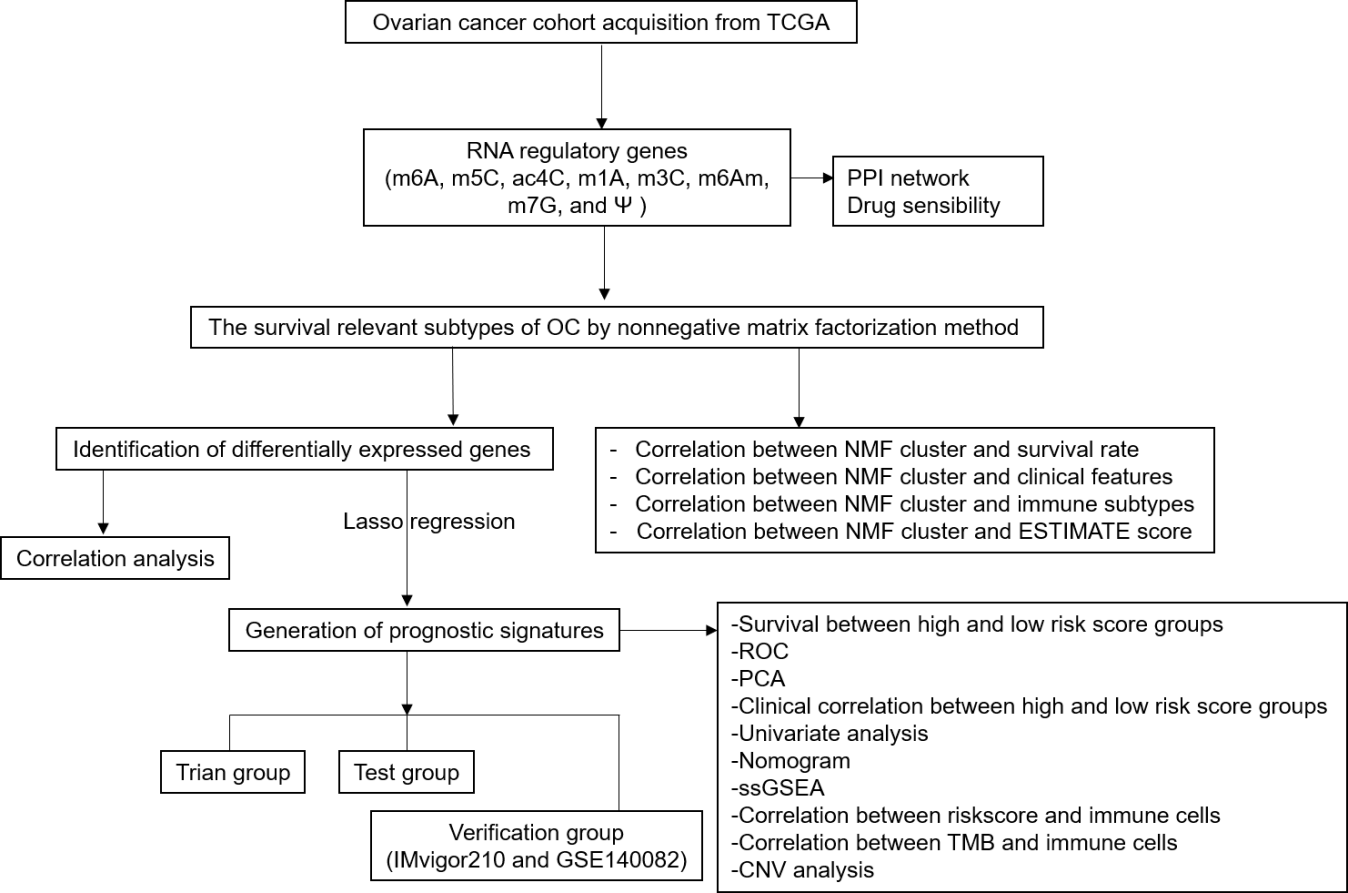
Flow chart for identification of RNA modification regulatory gene signature in ovarian cancer.

Our study classified 307 OC patients into 2 subtypes based on the expressions of 59 RRGs and identified 21 differentially expressed RRGs (DERRGs) between 2 subtypes. In previous studies, OC subtypes were clustered based on different types of genes using non-negative matrix factorization (NMF) method. According to 426 immune lncRNA pair data, OC samples could be classified into 2 molecular subtypes (36). Similarly, based on the expression profiles of 177 metabolism-related genes after a filtration of prognostic association, 3 different molecular subtypes of OC were obtained (37). Based on the immune cell infiltration in OC tumor microenvironment (TME), all OC samples were defined into 4 TME clusters, after which 2 genomic OC subtypes were identified according to differential expression genes among TME clusters (38). Another study reported that no more than 3 molecular subtypes should be classified for high-grade serous OC based on cross-population analysis (39). All these obtained subtype classifications were well effective, validated by their clinical feature correlation. Compared to them, this present study classified OC samples into 2 subtypes based on 59 RRGs expression profile, and also got a good consistency with clinical features. Then, lasso regression was used to construct a four-DERRG signature (ALYREF, ZC3H13, WTAP, and METTL1) model, which found the most valuable and critical regulators in m1A, m6A, m6Am, m5C, m7G, ac4C, m3C, and Ψ RNA modification processes. We conducted a comprehensive study of different types of RNA-modification regulators rather than only one specific RNA modification, which was more generally applicable to the evaluation of OC patients. The four-DERRG signature acted as an independent risk factor, which could be used in patient stratification, prediction, prevention, and immunotherapy targets of OCs. The research flow chart was presented for this study (**Figure 1**).

## Methods

### Data processing

In total, 307 OC patients were enrolled in this study, which contains both complete clinical information and expression data. The corresponding clinical features, including survival status, survival time, and progression-free-survival (PFS) time, were also downloaded from The Cancer Genome Atlas (TCGA) website (**Supplementary Table 1**). The mRNA expression level of 59 RRGs for different RNA modifications (m1A, m6A, m6Am, m5C, m7G, ac4C, m3C, and Ψ) was obtained from TCGA website (https://portal.gdc.cancer.gov/) (**Supplementary Table 2**), and those data were transformed with FPKM. The 59 RRGs included m6A regulators (KIAA1429, ZC3H13, METTL3, METTL14, WTAP, RBM15, RBM15B, FTO, ALKBH5, YTHDC1, YTHDC2, YTHDF1, YTHDF2, HNRNPC, IGF2BP1, IGF2BP2, YTHDF3, IGF2BP3, HNRNPA2B1, and RBMX), m5C regulators (TRDMT1, DNMT1, DNMT3A, DNMT3B, NSUN1, NSUN2, NSUN3, NSUN4, NSUN5, NSUN6, NSUN7, TET1, TET2, TET3, YBX1, and ALYREF), m1A regulators (TRMT6, TRMT61A, TRMT61B, TRMT10C, RRP8, ALKBH1, ALKBH3, YTHDF1, YTHDF2, YTHDF3, and YTHDC1), ac4C regulators (NAT10, and THUMPD1), m3C regulator METTL8, m6Am regulators (PCIF1, FTO, METTL3, and METTL4), m7G regulators (RNMT, METTL1, WDR4, and NUDT16), and Ψ regulators (PUS1, PUS3, PUS4, PUS7, PUS9, TRUB1, and TRUB2).

### The protein-protein interaction network and drug sensibility of RRGs

In order to evaluate the interactive associations of 59 RRGs for eight types of RNA modifications(m1A, m6A, m6Am, m5C, m7G, ac4C, m3C, and Ψ), their protein–protein interaction (PPI) network was mapped in the STRING database (https://string-db.org/) with combined score>0.9 (**Supplementary Table 3**).

The CellMiner (https://discover.nci.nih.gov/cellminer/) was used to evaluate the association between RRG expressions and drug sensibility. CellMiner is a genomic and drug analysis tool for exploring transcripts and drug paradigms of NCI-60 cell line set. NCI-60 cell line set, explored by National Cancer Institute’s (NCI) Developmental Therapeutics Program (DTP) in US, was used for anti-cancer drug screening and efficacy evaluation. Their correlation was validated in Corrplot R package with Spearman method (p < 0.05, and |Cor|>=0.4) on the basis of the relevant data obtained from CellMiner in OCs (**Supplementary Table 4**).

### Identification of OC subclasses

Based on these 59 RRGs, non-negative matrix factorization (NMF) clustering was analyzed subsequently. NMF clustering method was generally used to identify cancer molecular subtypes. Extracting biological correlation coefficient of data in gene expression matrix, NMF clustering could capture internal structural features to cluster cancer samples by organizing genes and samples. Target dataset was obtained *via* merging gene data and expression matrix after reading in them. During NMF clustering operation, different numbers of NMF subtypes were obtained. According to cophenetic value, the optimum clustering number was determined, based on which final grouping situation was settled. A filtration process was performed prior to NMF to exclude candidate genes whose median absolute deviation (MAD) values were low (MAD ≤ 0.5) in OC patients. The NMF R package (https://www.rdocumentation.org/packages/NMF/versions/0.23.0) was used to perform unsupervised NMF clustering (R version 4.1.1) on the metadata set, and the optimal cluster number 2 was selected as the coexistence correlation coefficient K value (**Supplementary Table 5**). Based on Kaplan–Meier method, the overall survival (OS) and PFS curves of OC subgroups were obtained using “survival” package in R (R version 4.1.1) software (https://www.bioconductor.org/packages/devel/bioc/vignettes/survtype/inst/doc/survtype.html).

### The correlation of clinical features in OC subclasses

The corresponding clinical features, including survival time, survival status, age at initial pathologic diagnosis, clinical stage, anatomic subdivision, radiation therapy, primary therapy outcome, histologic grade, lymphatic invasion, cancer status, and tumor residual disease, were also downloaded from TCGA website (**Supplementary Table 1**). Chi-square test (*X* ^2^) was used to analyze the correlation of clinical characteristics between NMF clusters 1 and 2, with statistical significance level of p <0.05. The clinical heatmap of 59 RRGs was performed with “pheatmap” R package (https://cran.r-project.org/web/packages/pheatmap/index.html) between different OC subclasses.

### The different immune subtype, and immune score between different NMF subtypes in OCs

OC samples were classified into four clusters, involving wound healing (Immune C1), IFN-gamma dominant (Immune C2), inflammatory (Immune C3), and lymphocyte depleted (Immune C4) based on immune model subtypes (**Supplementary Table 1**). Different immune subtype distribution between NFM clusters was analyzed with ggalluvial R package (https://www.rdocumentation.org/packages/ggalluvial/versions/0.12.3/topics/ggalluvial-package).

Based on expression data, stromal cells and immune cells in tissues of malignant tumor were estimated using an ESTIMATE algorithm. Based on specific biomarkers associated with stromal and immune cells infiltration in tumor samples, immune scores were estimated with ESTIMATE algorithm, which was derived from the public source website (https://sourceforge.net/projects/estimateproject/). The immune scores were calculated for each sample (**Supplementary Table 6**), and compared between different NFM subtypes in OCs. The violin-plots of ESTIMATE algorithm between different NFM subtypes were drawn with ggpubr R package (https://www.rdocumentation.org/packages/ggpubr/versions/0.4.0) with statistical significance level of p<0.05.

### Determination of DERRGs between different clusters in OCs

Unpaired student *t*-tests were used to calculate DERRGs of 59 RRGs between the NMF clusters 1 and 2 in OC patients, whose difference was statistically significant with adjusted p-value <0.05. Subsequently, Spearman method was used to perform the association between DERRGs by means of Corrplot R package (https://www.rdocumentation.org/packages/corrplot/versions/0.92) (p < 0.05).

### Establishment of DERRG signature in OCs with lasso regression

Lasso regression was a data processing tool, with the help of which prediction accuracy and rationality of the statistical model were enhanced *via* selection and regularization of variates. To obtain better performance parameters, variates were selectively put into the constructed model. Further, overfitting could be avoided *via* regularization of model complexity. The regularization degree of lasso regression was controlled by parameter lambda, and the penalty intensity of linear model with more variates was positively correlated with the value of lambda, based on which a model with fewer variates could be obtained. The prognostic model was constructed through identifying the relationship between the optimal selection of subset and lasso coefficient estimation. Lasso regression was performed based on DERRGs to identify the DERRG signature related to high risk of OCs, which was validated with the glmnet R package (https://www.rdocumentation.org/packages/glmnet/versions/4.1-4). The DERRG signature model identified by lasso regression calculated a riskscore associated with pathologically related clinical features for each OC tissue sample. Accordingly, 307 OC patients were randomly assigned to training and test groups, with each group assigned to high-risk and low-risk groups based on the median value of all riskscores (**Supplementary Table 7**). Further, measurements of riskscore-based classification were tested with receiver operating characteristic (ROC) curve and principal component analysis (PCA). The validity of the prognostic model was evaluated with Kaplan–Meier method in training and test groups.

To eliminate the over-fitting effect, the prognostic value of the DERRG signature was verified with two independent external validation cohorts, including cohorts imvigor210 (**Supplementary Tables 8, 9**) and GSE140082 (**Supplementary Table 10**). Imvigor210 study is a phase II clinical study using PD-L1 monoclonal antibody atezolizumab in one arm of locally progressive or metastatic tumor after platinum chemotherapy failure. Objective response rate (ORR) is primary end point, whereas PFS and OS are secondary end points. The response to immunotherapy based on imvigor210 cohort involved stable disease (SD), progressive disease (PD), complete response (CR), and partial response (PR) (**Supplementary Table 9**). GSE140082 cohort included 380 OC samples and corresponding integrated clinical follow-up information. The validity of the prognostic model in imvigor210 validation cohort and GSE140082 validation cohort was evaluated with Kaplan–Meier method.

Moreover, Cox regression was used to analyze clinical features related to OS in OC patients with univariate model. The clinical relevance of high-risk and low-risk groups was detected with pheatmap R package (http://bioconductor.org/packages/3.8/bioc/html/heatmaps.html). This riskscore evaluation nomogram was performed to assess the prognosis of OC patients including 1-, 3-, and 5-year survival rates, and then verified with decision-making tree method.

Gene-set enrichment analysis (GSEA) is a gratis software for analyzing genomic microarray data containing various functional gene sets. A total of 307 patients were classified into high-risk and low-risk groups based on OC riskscores. GSEA analysis was performed on TCGA data of the two groups to search for the significantly enriched gene sets in high-risk and low-risk groups (**Supplementary Table 11**).

### The correlations between riskscore or TMB or CNV and immune cells

CIBERSORT algorithm and LM22 gene signature were used to determine the proportions of different immune cells in OCs, which allow for identifying 22 types of human immune cell phenotypes with high sensitivity and specificity. Preparation of gene expression profiles was performed with standard annotation files, and corresponding data were submitted to the CIBERSORT website (http://cibersort.stanford.edu/), in which LM22 signature and 1,000 permutations were used to run the algorithm (**Supplementary Table 12**). Corrplot R package was used to analyze the correlation between immune cells and riskscore (https://www.rdocumentation.org/packages/corrplot/versions/0.92) with Spearman method (p < 0.05), encompassing monocytes, macrophages M1, macrophages M2, macrophages M0, eosinophils, neutrophils, mast cells activated, mast cells resting, dendritic cells activated, dendritic cells resting, NK cells activated, NK cells resting, T cells regulatory (Tregs), T cells follicular helper, T cells gamma delta, B cells naïve, plasma cells, B cells memory, T cells CD4 naïve, T cells CD4 memory activated, T cells CD4 memory resting, and T cells CD8.

Apart from this, the Level 3 RNA-seq data of immune checkpoints were selected from TCGA database (https://portal.gdc.cancer.gov/). Different levels of immune checkpoints were analyzed between different methylation subtypes in OCs with unpaired student *t*-tests, including VTCN1, PDCD1, CTLA4, CD276, CD80, CD274, PDCD1LG2, and CD86.

TMB scores were generated with Maftools R package (**Supplementary Table 13**). The relevance of TMB and immune cells was validated with Corrplot R package (https://www.rdocumentation.org/packages/corrplot/versions/0.92) with Spearman method (p < 0.05).

Copy number variant (CNV) data were based on UCSC Xena datasets (https://xenabrowser.net/datapages/) in **Supplementary Table 14**. The correlation between identified gene expression in LASSO model and CNV was calculated with Kruskal test (p < 0.05), and boxplots were plotted with barplot R package (https://www.rdocumentation.org/packages/graphics/versions/3.6.2/topics/barplot).

### Statistical analysis

For variables following a normal distribution, unpaired student *t*-test was used to calculate the p value, and p < 0.05 was set as the level of statistical significance. Survival curves were generated with the Kaplan-Meier method, and statistical significance of differences was evaluated through the Log-rank (Mantel-Cox) test, in which p < 0.05 represented differences were statistically significant. The hazard ratio of univariate Cox proportional hazard regression model was established with statistical significance of p<0.05. We also shared the code that was used for this study in a public repository-GitHub (https://github.com/peixianzheng/OCmodel.git).

## Results

### Ovarian cancer subtypes based on RRGs with NMF

In total, 59 RRGs for RNA modifications (m1A, m6A, m6Am, m5C, m7G, ac4C, m3C, and Ψ) were obtained from TCGA website (**Supplementary Table 2**), including m6A regulators (KIAA1429, ZC3H13, METTL3, METTL14, WTAP, RBM15, RBM15B, FTO, ALKBH5, YTHDC1, YTHDC2, YTHDF1, YTHDF2, HNRNPC, IGF2BP1, IGF2BP2, YTHDF3, IGF2BP3, HNRNPA2B1, and RBMX), m5C regulators (TRDMT1, DNMT1, DNMT3A, DNMT3B, NSUN1, NSUN2, NSUN3, NSUN4, NSUN5, NSUN6, NSUN7, TET1, TET2, TET3, YBX1, and ALYREF), m1A regulators (TRMT6, TRMT61A, TRMT61B, TRMT10C, RRP8, ALKBH1, ALKBH3, YTHDF1, YTHDF2, YTHDF3, and YTHDC1), ac4C regulators (NAT10, and THUMPD1), m3C regulator METTL8, m6Am regulators (PCIF1, FTO, METTL3, and METTL4), m7G regulators (RNMT, METTL1, WDR4, and NUDT16), and Ψ regulators (PUS1, PUS3, PUS4, PUS7, PUS9, TRUB1, and TRUB2).

**Figure 2:**
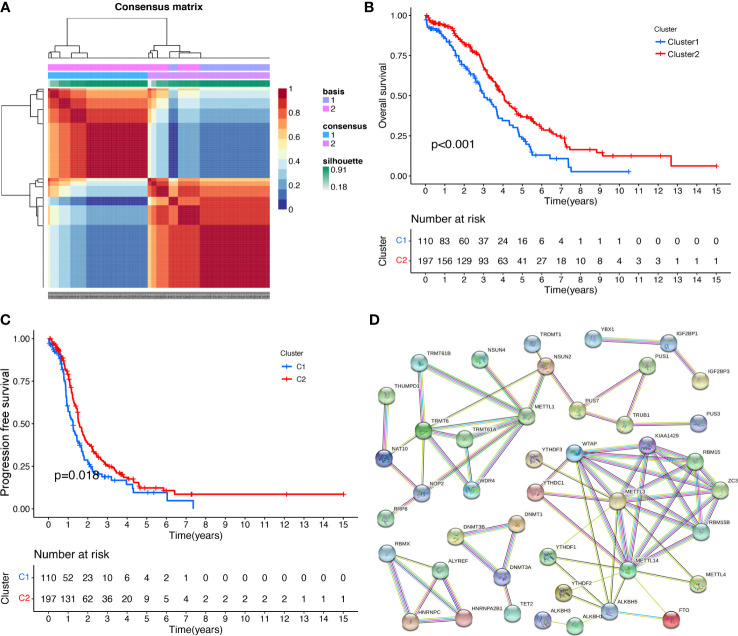
The RNA modification subtypes based on NMF analysis. **(A)** Clustering heat map of samples at consensus k = 2. Different colors reflect different cluster numbers; the color gradient is from white to blue, indicating the consensus of progression. **(B)** The OS analysis between two RNA-modification subtypes in ovarian cancer. **(C)** The PFS analysis between two RNA modification subtypes in ovarian cancer. **(D)** The PPI network of RNA-modification regulatory genes.

NMF method is an efficient tool for dimensionality reduction of cancer subtype identification. In this study, the best value of clusters number (K) was obtained using factoextra package. When K was equal to 2, the OC samples (n=307) were classified into two distinct subtypes (Cluster 1: n=110; Cluster 2: n=197) by NFM method (**Figure 2A**; **Supplementary Table 5**), showing a favourable match between OC samples and their identified subtypes. It is worth noting that OC patients in Cluster 1 showed fine OS status, whereas Cluster 2 patients displayed poor prognosis (**Figure 2B**). Meanwhile, OC patients in Cluster 1 showed good PFS rate, whereas Cluster 2 had poor prognosis (**Figure 2C**; **Supplementary Table 1**).

### The significant PPI network of RRGs and the drug sensibility

Protein–protein interaction analysis was performed on 59 RRGs with STRING. The spectrum of nodes combined scores was from 0.900 to 0.999 (**Figure 2D**; **Supplementary Table 3**). Some protein–protein interactions showed high combined scores (>0.999), such as ZC3H13 and KIAA1429, WTAP and KIAA1429, METTL14 and KIAA1429, KIAA1429 and METTL3, METTL1 and WDR4, KIAA1429 and METTL14, METTL14 and METTL3, WTAP and METTL14, METTL3 and KIAA1429, WTAP and METTL3, METTL3 and METTL14, RBM15 and WTAP, TRMT6 and TRMT61A, TRMT61A and TRMT6, WDR4 and METTL1, ZC3H13 and WTAP, KIAA1429 and WTAP, WTAP and METTL3, WTAP and RBM15, METTL14 and WTAP, KIAA1429 and ZC3H13, and ZC3H13 and WTAP.

**Figure 3:**
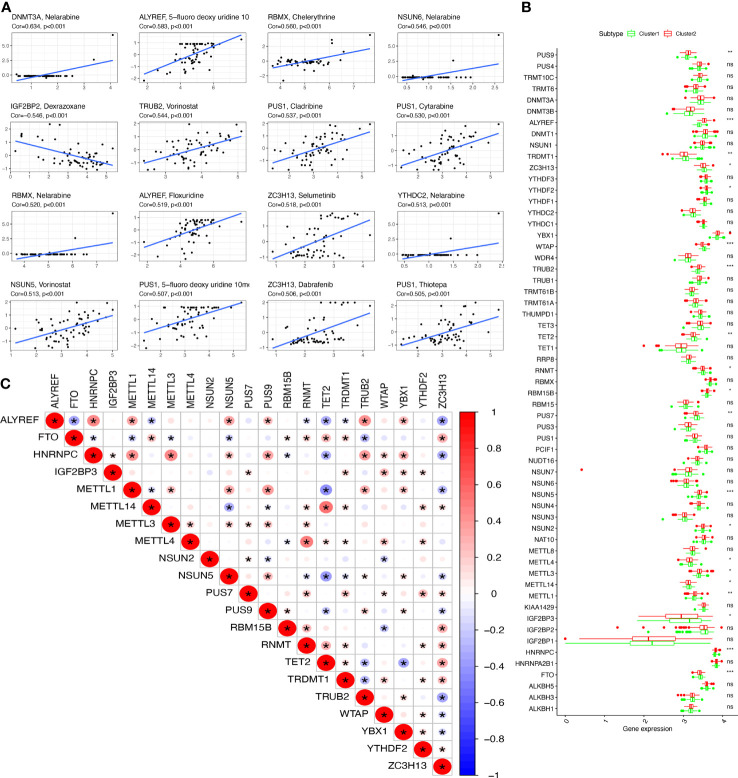
The drug sensitivity and DERRGs. **(A)** The drug sensitivity of DERRGs. **(B)** DERRGs between two RNA-modification subtypes. **(C)** Correlation analysis for DERRGs. *p < 0.05, **p < 0.01, and ***p < 0.001. ns, no significance.

Some RRGs showed significant associations with drug sensibility, with |correlation coefficient|>0.5 and p<0.05 (**Supplementary Table 4**), such as PUS1 and triethylenemelamine, PUS1 and thiotepa, ZC3H13 and dabrafenib, PUS1 and 5-fluoro deoxy uridine 10mer, NSUN5 and vorinostat, YTHDC2 and nelarabine, ZC3H13 and selumetinib, ALYREF and floxuridine, RBMX and nelarabine, PUS1 and cytarabine, PUS1 and cladribine, TRUB2 and vorinostat, NSUN6 and nelarabine, RBMX and chelerythrine, ALYREF and 5-fluoro deoxy uridine 10mer, DNMT3A and nelarabine, IGF2BP2 and dexrazoxane, IGF2BP2 and SR16157. Some of them were plotted (**Figure 3A**).

### DERRGs between clusters 1 and 2 of OCs

The DERRGs of 59 RRGs were calculated with adjusted p-value <0.05, from which 21 DERRGs were identified, including PUS9, ALYREF, TRDMT1, ZC3H13, YTHDF2, YBX1, WTAP, TRUB2, TET2, RNMT, RBM15B, PUS7, NSUN5, NSUN2, METTL3, METTL4, METTL14, METTL1, IGF2BP3, HNRNPC, and FTO (**Figure 3B**). Subsequently, the association between DERRGs was evaluated using Corrplot with Spearman method (p < 0.05). Some of them showed high correlation coefficient, including METTL14 and TET2, METTL1 and TET2, HNRNPC and METTL1, HNRNPC and METTL3, HNRNPC and ALYREF, ALYREF and TRUB2, METTL4 and RNMT (**Figure 3C**).

### Correlation between OC subtypes and clinical characteristics or immune

**Figure 4:**
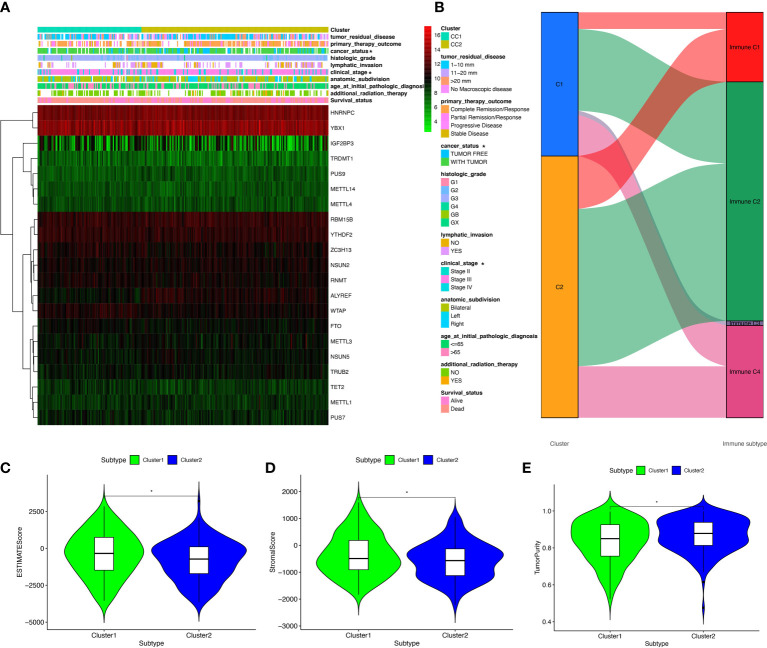
The clinical heatmap and immune status between two RNA-modification subtypes. **(A)** The clinical heatmap between two RNA-modification subtypes. **(B)** The immune types between two RNA-modification subtypes. **(C)** StromalScore between two RNA-modification subtypes. **(D)** ESTIMATEScore between two RNA-modification subtypes. **(E)** TumorPurity between two RNA-modification subtypes. *p < 0.05.

Clinical information was obtained from TCGA database, including age (from 30 to 84 years), survival status (alive and dead), anatomic subdivision (left, bilateral and right), follow-up outcome (complete remission/response, partial remission/response, stable disease, and progressive disease), pathologic stage (stages I, II, III, and IV), cancer status (with tumor or tumor-free), lymph node metastasis (yes/no), radiation therapy(yes/no), histologic grade (G1-G3), and tumor residual disease (No macroscopic disease, 1-10 mm, 11-20 mm, and >20 mm) (**Supplementary Table 1**). Further, the correlation between clinical characteristics and OC subtypes was explored. Some clinical characteristics, such as pathologic stage and cancer status, were significantly associated with OC subtypes (**Figure 4A**).

Additionally, the correlation between OC subtypes and immune was also analyzed, including immune type (**Supplementary Table 1**) and immune-related scores (**Supplementary Table 6**). Immune type correlation analysis showed that, in Cluster 1, 11 samples were enriched in immune type C1, 52 samples in immune type C2, 3 samples in immune type C3, and 27 samples in immune type C4; whereas, in Cluster 2, 35 samples were enriched in immune type C1, 104 samples in immune type C2, and 34 samples in immune type C4. Clusters 1 and 2 showed significant different distribution among different immune types (**Figure 4B**). In terms of immune-related scores, Cluster 1 showed higher StromalScore and ESTIMATEScore, and lower TumorPurity compared to Cluster 2 (**Figures 4C–E**).

### Construction of riskscore model based on four DERRGs

**Figure 5:**
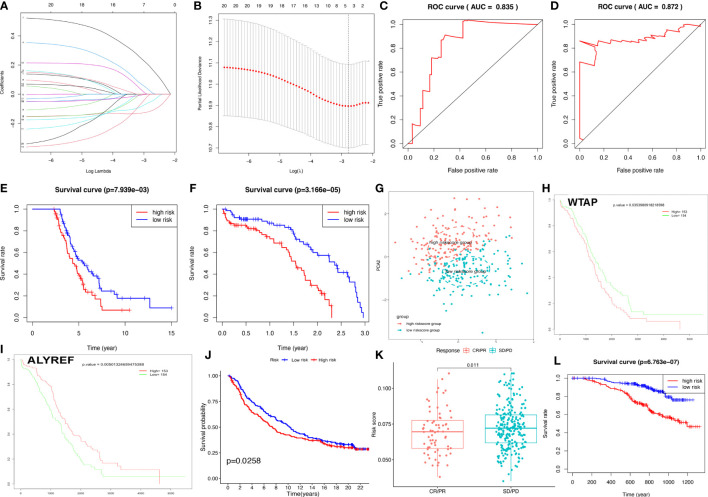
Construction of riskscore model based on four DERRGs. **(A, B)**. Lasso regression identified the prognostic model in ovarian cancer. **(C)** ROC analysis between high- and low-riskscore groups in training group. **(D)** ROC analysis between high- and low-riskscore groups in test group. **(E)** OS analysis between high- and low-riskscore groups in training group. **(F)** OS analysis between high- and low-riskscore groups in test group. **(G)** PCA analysis between high- and low-riskscore groups in ovarian cancer. **(H)** OS analysis of WTAP. **(I)** OS analysis of ALYREF. **(J)** OS analysis between high- and low-riskscore groups in imvigor210 cohort. **(K)** The response for immunethreapy based on imvigor210 cohort showed stable disease (SD), progressive disease (PD), complete response (CR), and partial response (PR). **(L)**. OS analysis between high- and low-riskscore groups in GSE140082 cohort.

The OC samples were randomly divided into training (n=155) and test groups (n=152). Training and test groups were divided into high-risk and low-risk score groups according to the riskscores based on 21 DERRGs (**Supplementary Table 7**). A set of four DERRGs (ALYREF, ZC3H13, WTAP, and METTL1) were found to increase the risk of poor prognosis in OCs based on Lasso regression analysis (**Figures 5A, B**; **Supplementary Table 7**), when log (lambda) was set between −2 and −3. Thus, the obtained risk scoring formula was as follows: risk score = -0.116404020427426*ALYREF + 0.0203573242796506*ZC3H13 + 0.186320163255671*WTAP + -0.0528745603956501*METTL1. Per the ROC curve, area under the curve (AUC) was equal to 0.835 in training group, and ROC curve showed AUC= 0.872 in test group (**Figures 5C, D**). OS analysis was performed with Kaplan–Meier method between high-risk and low-risk score groups in the training and test clusters, respectively. Overall survival rate was significantly different (**Figures 5E, F**). Validated by PCA, it is observed that the whole OC samples were well classified into high-risk and low-risk groups based on riskscores (**Figure 5G**). Among the identified DERRGs in prognosis model, ALYREF and WTAP individually was significantly related to OS (**Figures 5H, I**). The constructed riskscore model based on 4 DERRGs was also verified by two independent external validation cohorts (**Supplementary Tables 8–10**). The imvigor210 cohort showed that the prognosis of high-riskscore group was poorer than that of low-riskscore group (**Figure 5J**). The response to immunotherapy based on imvigor210 cohort showed that PD and SD had high riskscores, whereas PR and CR had low riskscores (**Figure 5K**). Additionally, the GSE140082 cohort showed that the prognosis of high-riskscore group was poorer than that of low-riskscore group too (**Figure 5L**).

**Figure 6:**
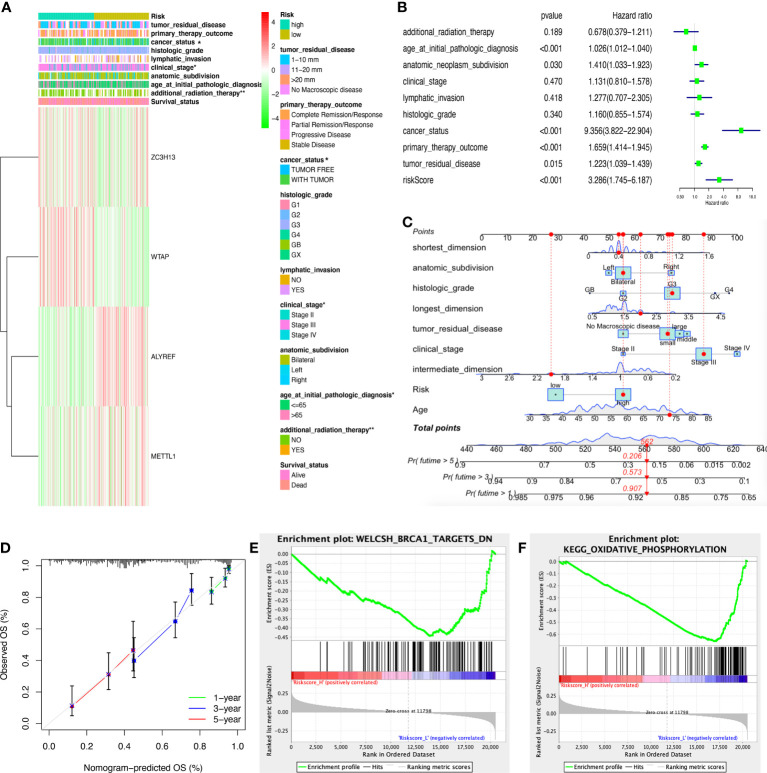
Clinical correlation between high- and low-riskscore groups. **(A)** The clinical heatmap between high- and low-riskscore groups. **(B)** The univariate Cox regression analysis of risk factors in ovarian cancer. **(C)** The risk score assessment nomogram to evaluate prognosis in ovarian cancer (1-, 3-, and 5-year survival rates). **(D)** The decision-making tree plot of nomogram. **(E)** GSEA plot of WELCSH_BRCA1_TARGETS_DN between high and low riskscore groups. **(F)** GSEA plot of KEGG_OXIDATIVE_PHOSPHORYLATION between high and low riskscore groups. *p < 0.05 and **p < 0.01.

The heatmap illustrated that the riskscore group had a significant connection with clinical characteristics, including age at initial diagnosis, cancer status, pathologic stage, and radiation therapy (**Figure 6A**). The univariate Cox regression analysis found that OS was significantly correlated with age at initial pathologic diagnosis, cancer status, anatomic subdivision, tumor residual disease, primary therapy outcome, and riskscore (**Figure 6B**). Furthermore, the nomogram was drawn to predict the survival rate (1, 3, 5 year) of OC patients based on basic clinical features and riskscore (**Figure 6C**). The decision-making tree plot verified that nomogram could provide good effect (**Figure 6D**).

The ssGSEA was executed between high- and low-riskscore groups to show the different gene sets. A total of 44 significant gene sets have been enriched (**Supplementary Table 11**). The gene sets were significantly enriched in WELCSH BRCA1 TARGETS DN, PENG GLUTAMINE DEPRIVATION DN, REACTOME PROCESSING OF CAPPED INTRONLESS PRE MRNA, BONOME OVARIAN CANCER POOR SURVIVAL DN, WONG EMBRYONIC STEM CELL CORE, KEGG OXIDATIVE PHOSPHORYLATION, LU EZH2 TARGETS UP, etc, between high and low riskscore groups (**Figure 6E, F**; **Supplementary Table 11**).

### The four-DERRG signature-based riskscores were significantly correlated with immune and TMB and CNV

**Figure 7:**
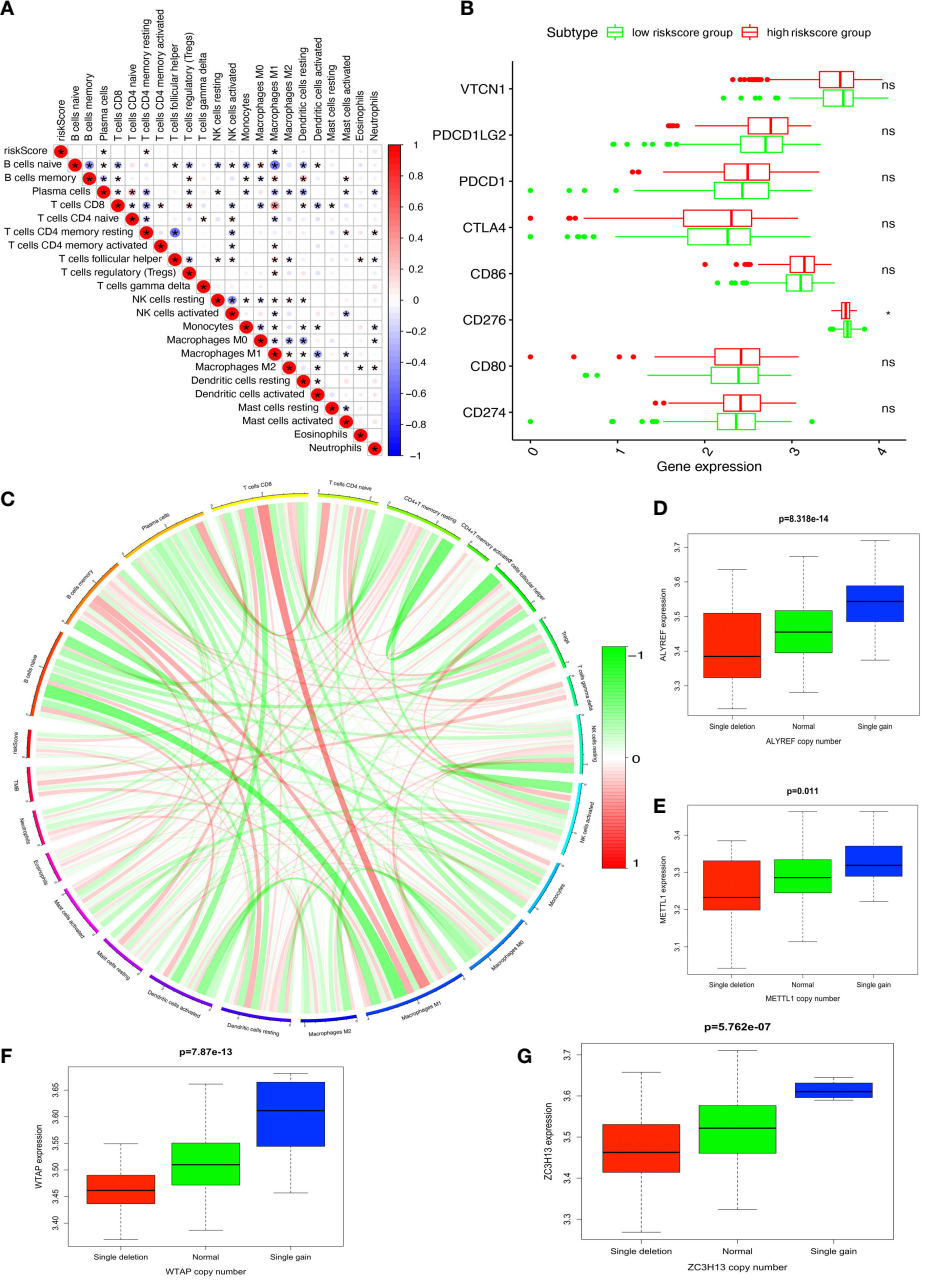
Immune and TMB between high- and low-riskscore groups. **(A)** The correlation between riskscore and immune cells. **(B)** The correlation between riskscore and immune check points. **(C)** The correlation between TMB and immune cells. **(D)** The cor-relations between mRNA expression and CNV alteration frequency of ALYREF in ovarian cancer. **(E)** The cor-relations between mRNA expression and CNV alteration frequency of ZC3H13 in ovarian cancer. **(F)** The cor-relations between mRNA expression and CNV alteration frequency of WTAP in ovarian cancer. **(G)** The cor-relations between mRNA expression and CNV alteration frequency of METTL1 in ovarian cancer. *p < 0.05, **p < 0.01, and ***p < 0.001. ns, no significance.

The four-DERRG signature-based riskscores were positively correlated with CD4+ memory resting T cells, and negatively correlated with macrophages M1 and plasma cells (**Figure 7A**, **Supplementary Table 12**). Additionally, immune checkpoints also showed significant differences between these high- and low-riskscore subtypes (**Figure 7B**), such as CD276. The TMB was positively correlated with Macrophages M1, T cells gamma delta, B cells memory, and showed negative correlation with NK cells activated, and B cells naïve (**Figure 7C**; **Supplementary Table 13**).

CNV was the repeated sections of the genome that varied between individuals. Whether the CNV affected the expression of identified genes in LASSO model (ALYREF, ZC3H13, WTAP, and METTL1), the expression perturbations of identified genes were therefore explored (**Supplementary Table 14**). The CNV alteration frequencies of those genes were widespread positively correlated with the expressions of those genes (**Figures 7D–G**).

## Discussion

### Role of RNA modification and its regulation in OCs

More than 170 diverse types of post-transcriptional modifications were detected to be emerged in RNAs. All these modifications could occur in ribose and four RNA bases, and all RNA species could be modified, especially transfer RNAs (tRNAs) and ribosomal RNAs (rRNAs) (40). Much evidence suggested that dysregulation of the RNA epigenetic pathways played a crucial role in pathogenesis of many human cancers (9). It is known that RNA modification process was dynamic, which helped cells promptly adapt to changes in the microenvironment (41). The capability of adapting the changes of microenvironment played a crucial role in survival of tumor cells, suggesting that RNA modification was vital in cancer (10). Cancer was defined as a disease featured by the progressive accumulation of genetic and epigenetic changes in diverse oncogenes as well as tumor suppressor genes. Meanwhile, a growing number of studies have showed that epitranscriptomics played an important part in the pathological process. RNA modifications have been proved to be crucial regulators of cancer (9). Abnormal expressions of RNA modification regulators were functionally associated with cell proliferation, cell differentiation, cell self-renewal, invasion, stress adaptation, treatment resistance, and survival; and all of them were important features in cancer (10). For instance, in liver cancer, YTHDF2 promoted the phenotype of cancer stem cell and cancer metastasis through regulation in m6A methylation of pluripotency factor OCT4 mRNA (42). In bladder cancer, ALYREF was proved to strengthen the stability of PKM2 mRNA and bind to m5C sites of specific regions. ALYREF high expression increased cancer cell proliferation *via* glycolysis reaction mediated by PKM2 (43). Also in bladder cancer, ac4C modification mediated by NAT-10 has been certified to increase bladder cancer progression (44). Additionally, in lung cancer, m7G tRNA modifications mediated by METTL1/WDR4 were found to play a crucial role in regulation of mRNA translation process and cancer progression (45). In OCs, m6A modifications mediated by FTO restrained cancer stem cells self-renewing process through inhibition of cAMP signaling (46). This present study further demonstrated the significance of RNA modification along with its regulation in cancers involving OCs.

### Role of RNA modification and its regulation in immune microenvironment and immunotherapy of OCs

RNA modifications and its regulation were closely associated with immune microenvironment in OCs and other types of tumors, including immune molecules, immune cells, and immune pathways. Recent studies revealed that RNA modifications regulated activation of immune cells and their infiltration in tumor microenvironment, and afterwards influenced the immunotherapy outcomes. In consequence, RNA modifications had great value as tumor immunotherapy targets (47). A study found that ALKBH5, an important m6A demethylase, regulated PD-L1 expression in intrahepatic cholangiocarcinoma. ALKBH5 suppressed enlargement and cytotoxicity of T cells through preserving PD-L1 expression. Moreover, ALKBH5 played a complex part in tumor immune microenvironment, mainly manifested in overexpression of PD-L1 on mononuclear macrophage and reduced infiltration of myeloid-derived suppressor-like cells (48). Another study revealed positive correlation between m6A writer METTL3 expressions and effector molecules in natural killer (NK) cells. The homeostasis of NK cells was changed with loss of METTL3 in NK cells, and infiltration and function of NK cells were inhibited in tumor microenvironment, which resulted in increasing rate of tumor growth and reduced survival time in mice. The protein expression level of SHP-2 modified by m6A regulators was decreased in METTL3-deficient NK cells. IL-15 response was decreased with reduced SHP-2 activity in METTL3-deficient NK cells, which was related to inhibition of activating AKT and MAPK signaling pathways (49). In addition, a study reported overexpression of circIGF2BP3 was negatively correlated with CD8+ T cells infiltration in non-small cell lung cancer, which functionally compromised antitumor immunity in immunodeficient mice. METTL3 mediated circIGF2BP3 m6A modification and promoted its circulation *via* YTHDC1. CircIGF2BP3 disrupted cancer immune response through upregulating PKP3 expression *via* miR-328-3p and miR-3173-5p. Further, PKP3 strengthened the stability of OTUB1 mRNA through binding to the RNA-binding protein FXR1, which increased PD-L1 enrichment through promoting deubiquitination. The deletion of PD-L1 in tumor entirely interrupted the effect of circIGF2BP3/PKP3 axis on response to CD8+ T cells. CircIGF2BP3/PKP3 inhibition increased the efficacy of anti-PD-1 treatment in lung cancer mouse model (50). In terms of ovarian OCs, a study demonstrated m1A modifications played critical roles in tumor immune microenvironment formation and prognosis of OC patients (51). Identically, m6A modification was proved to play an essential part in tumor microenvironment cell infiltration in OCs (52). This present study further analyzed the relationship of OC subtypes and immune types. The results showed obviously different distribution of immune types in different clusters, indicating immune molecules, immune cells, or immune pathways involved in different OC subtypes may be different. Additionally, we constructed four-DERRG signature model to calculate riskscores of OC patients and found it was positively correlated with CD4+ memory resting T cells, and negatively correlated with plasma cells and macrophages M1, which suggested ones to pay more attention to these three types of immune cells and their potential target functions in OC immunotherapy.

### Role of identified RNA regulator genes and significance of related drug sensibility in OCs

In total, 59 RRGs were identified in this study, most of which were proved to be associated with OC pathogenesis in previous studies. DNMT1 was a key RRG in chemotherapy resistance of OCs, and the feedback regulation between DNMT1 and miR-30a/c-5p played an important part in epithelial-mesenchymal transition and cisplatin-resistance (53). Similarly, overexpression of miR-185 or miR-152 inhibited cell proliferation and promoted apoptosis to increase drug sensibility to cisplatin through suppressing DNMT1 directly in OCs (54). Another study reported that ubiquitin-conjugating enzyme E2 N regulated paclitaxel sensibility of OC cells *via* DNMT1-CHFR-Aurora A pathway (55). A transcriptome m6A methylation analysis towards endometrioid ovarian cancer showed the influence of METTL3 on endometrioid ovarian cancer, and revealed the knockout of METTL3 resulted in distinct decrease of proliferation, increasing apoptosis, and G0/G1 blocking of cell cycle (56). Other studies proved that METTL3 increased OC progression and promoted invasion *via* epithelial-mesenchymal transition and AXL translation (57), and accelerated tumorigenesis and metastasis through suppressing CCNG2 expression targeting miR-1246 in OC (58). Furthermore, another study illustrated the important role of METTL3 in mediating miR-126-5p maturation and promoting OC progression *via* PI3K/Akt/mTOR pathway (59). A meta-analysis suggested that METTL3 upregulation was significantly associated with poor prognosis of OC patients (60). TBX1 was a prognostic marker of multidrug resistance and cancer progression. Nuclear YBX1 expression level might be an independent factor of poor prognosis in OCs (61), and YBX1 nuclear translocation was regulated by Akt activation, influencing drug resistance genes expression in OC cells (62). YBX1 inhibition might contribute to reduction of cancer progression, antagonism of treatment resistance, and decrease of OC patient mortality (63). IGF2BP1 strengthened aggressiveness of OC cells through antagonizing miRNA-impaired gene expression (32), and enhanced invasive growth of OC cells driven by SRC/MAPK (64). DNMT3A promoted Warburg effect *via* miR-145 in OC cells (65). Double negative feedback of miR-29b and DNMT3A/3B promoted OC progression (29). Feedback between DNMT3A and miR-143 was a critical epigenetic regulator of cisplatin resistance in OCs (66). WTAP acting as an oncogenic factor promoted OC progression *via* WTAP-HBS1L/FAM76A axis (67). WTAP was highly expressed in high-grade serous OCs. WTAP overexpression was significantly related to lymphatic metastasis, whereas down regulation of WTAP contributed to weakness of cell proliferation as well as migration, and increased apoptosis in OC cell lines (68). ALKBH5 suppressed autophagy and enhanced proliferation and invasion *via* BCL-2 and miR-7 in epithelial ovarian cancer (69). Tumor growth and resistance to cisplatin were promoted *via* ALKBH5-HOXA10 loop through mediating JAK2/STAT3 signaling pathway in epithelial ovarian cancer (70). A multi-omics analysis of OCs showed that YTHDF1 promoted translation of EIF3C through combining with EIF3C mRNA modified by m6A and simultaneously promoted the whole output of translation to accelerate the OC tumorigenesis and metastasis (71). Knockdown of YTHDF1 suppressed cancer stem cell-like characteristics in OC cells resistant to cisplatin (72). TET1 inhibited Wnt/β-catenin signaling pathway through demethylating and upregulating SFRP2 and DKK1, two upstream antagonists in this pathway, to suppress cell metastasis and epithelial-mesenchymal transition in OCs (73). TET1 expression was related to not only low survival rate of terminal epithelial ovarian carcinoma, but migration, growth, stemness, and tumorigenicity of OC cells (74). TET1 expression also resulted in cisplatin resistance targeting vimentin in OCs (75). A study found that TET2 was significantly correlated with tumor-related fibroblast infiltration in OCs (76). IGF2BP2 increased aggressiveness and stemness by upregulating circ_0000745 *via* a miR-3187-3p/ERBB4/PI3K/AKT axis in OC cells (77). HNRNPC and nuclear factor I X were targeted by miR-744-5p in inducing apoptosis of OC cells (78). HNRNPA2B1 promoted OC malignant phenotype by upregulating expression of Lin28B (31). YTHDF2 distinctly accelerated cell proliferation and metastasis in epithelial ovarian cancer cell lines, and its overexpression reversed the decrease of cell proliferation and migration of epithelial ovarian cancer mediated by miR-145 (79). YTHDC2 was verified to play a key part in controlling meiosis in human, within which pathogenic variants were related to primary ovarian insufficiency (80). NAT10 was involved in tubulin processing, associated with cell growth in epithelial ovarian cancer (81). METTL14 overexpression inhibited cell proliferation of OC through suppressing expression of TROAP based on m6A RNA methylation (82). IGF2BP3 overexpression inhibited cancer cell apoptosis. The volume of tumors decreased and cancer metastasis indicator proteins were downregulated after treated with IGF2BP3 siRNA in ovarian clear cell carcinoma (83). Knockdown of IGF2BP3 reduced cell proliferation, invasion and migration, and enhanced platinum sensibility through increasing hCTR1 expression in OC cells, a copper transporter taking part in platinum uptake (33). FTO inhibited self-renewing of stem cells in OC and tumorigenesis *via* cAMP signaling pathway (46). Overexpression of FTO significantly promoted viability and autophagy, but reduced apoptosis in OCs (30). A bioinformatics analysis suggested that PUS7 was a potential marker for diagnosis and target for OC treatment (84). TET3 blocked epithelial-mesenchymal transition induced by TGF-β1 through demethylating miR-30d precursor gene promoter to suppress OCs (85). TRDMT1 overexpression decreased cisplatin sensibility and TRDMT1 inhibitor could reverse this change (86). TRMT10C silencing inhibited cell proliferation, migration and clone formation in OCs (87). These research results demonstrated that RRGs played crucial roles in OC biological behaviors and clinical characteristics. Further, RRGs were potential therapeutic targets in OC treatment strategies.

In previous study, many RRGs were certified to associate with drug sensibility or drug resistance in OCs, such as DNMT1 and cisplatin (53, 54), DNMT1 and paclitaxel (55), DNMT3A and cisplatin (66), ALKBH5 and cisplatin (70), TET1 and cisplatin (75), IGF2BP3 and platinum (33), and TRDMT1 and cisplatin (86). Similarly, this present study also found some RRGs were significantly associated with different types of drug sensibility in OCs, such as PUS1 and triethylenemelamine, PUS1 and thiotepa, ZC3H13 and dabrafenib, PUS1 and 5-fluoro deoxy uridine 10mer, NSUN5 and vorinostat, YTHDC2 and nelarabine, ZC3H13 and selumetinib, ALYREF and floxuridine, RBMX and nelarabine, PUS1 and cytarabine, PUS1 and cladribine, TRUB2 and vorinostat, NSUN6 and nelarabine, RBMX and chelerythrine, ALYREF and 5-fluoro deoxy uridine 10mer, DNMT3A and nelarabine, IGF2BP2 and dexrazoxane, and IGF2BP2 and SR16157. Vorinostat, one kind of histone deacetylase inhibitor, has been validated to play a role in multiple tumor treatments, such as melanoma (88), malignant glioma (89), and glioblastoma (90), in a RNA modification regulation manner. Chelerythrine, extracted from four plants of families Rutaceae and Papaveraceae, was one type of plant active ingredient with diverse functions involving anti-inflammation, analgesia, anti-bacteria and anticancer (91). Cladribine, a chlorodeoxyadenosine, acted as the first line treatment of hairy cell leukemia, and it could also be used in the drug therapies of adult systemic mastocytosis and multiple sclerosis (92–94). Cytarabine was one of the most crucial chemotherapy drugs in acute myeloid leukemia, which was usually combined with daunorubicin (95). Dabrafenib was an inhibitor of BRAF kinase, which could be used solely to treat unresectable or metastatic melanoma with BRAF V600E mutation, and to treat BRAF V600E or V600K mutated melanoma combined with trametinib (96). Similarly, for anaplastic thyroid cancer with BRAF V600E mutation, dabrafenib was also recommended together with trametinib (97). Dexrazoxane was an antidote for anthracycline chemotherapy extravasation approved by Food and Drug Administration (FDA), with a prominent cardioprotectant role in anthracycline-induced cardiotoxicity when treating cancers such as breast cancer (98–100). Floxuridine was a pyrimidine analogue routinely applied in colorectal cancer liver metastases management, progressively evolving as the superior drug for hepatic arterial infusional chemotherapy (101). Nelarabine, a synthetic antineoplastic compound targeted to T cell lymphoblastic leukemia and lymphoma, was an effective drug to treat pediatric and adult T cell acute lymphoblastic leukemia and lymphoma (102). Selumetinib, a highly specific inhibitor of mitogen activated protein kinase 1 and 2, was mainly used in treatments of neurofibromas related to neurofibromatosis type 1, pediatric low-grade gliomas, non-small cell lung cancer, and melanoma (103). SR16157 was a steroid sulfatase inhibitor and also a selective estrogen receptor α modulator, which has been used in clinical trials of breast cancer (104). Triethylenemelamine, owning a nitrogen mustardlike effect, was a crucial chemotherapy agent useful in the management of diverse neoplastic diseases, such as Hodgkin’s disease, malignant lymphoma, and chronic lymphocytic leukemia (105). Thiotepa was an alkylating agent used in the treatment of breast cancer, ovarian cancer, and bladder cancer currently (106–108). This finding gave ones a deep insight to understand the relationship between RRGs and drug sensibility. Meanwhile, it provided clues to explore the mechanism of drug sensibility change that is regulated at the RNA modification level, and opened up various novel possibilities in OC treatment strategies.

### Significance of four-DERRG signature model and differential signaling pathway

Among 21 DERRGs, four DERRGs (ALYREF, ZC3H13, WTAP, and METTL1) that were significantly associated with poor prognosis in OCs were selected to construct a four-DERRG signature model with Lasso regression analysis. Based on the established risk scoring formula, one could calculate riskscore of every OC sample with high accuracy, and then all OC patients were classified into high-risk and low-risk groups according to the mean values of their riskscores. This study found that overall survival rate was connected with subgroups both in training and test groups, indicating that overall survival rate of OC patients could be forecasted based on this riskscore model. External validation cohort results were also consistent with internal ones, and further suggested this riskscore model can be applicable for assessment of immunotherapy response and prognosis in OC patients. Additionally, a significant correlation between clinical features and risk groups was discovered, including age at initial diagnosis, clinical stage, cancer status, and radiation therapy, which suggested that potential initial diagnostic time, clinicopathological typing of tumors, tumorigenesis, and effectiveness of treatment strategies in OCs could be estimated based on this risk model. Moreover, riskscore was found to act as an independent hazard factor for overall survival rate of OC patients. Thus, this present study provided a succinct and clear method to estimate the patient survival rate of one-, three- and five-year, in which shortest dimension, longest dimension, intermediate dimension, anatomic subdivision, histologic grade, tumor residual disease, clinical stage, age, and riskscore were involved. It provided ones a novel pattern to score the prognosis of OC patients, which would contribute to patient stage grading and clinical treatment.

Among four-DERRGs (ALYREF, ZC3H13, WTAP, and METTL1) in the prognosis model, WTAP has been widely studied in OCs. WTAP acting as an oncogenic factor was related to cell proliferation, migration, cancer progression, and lymphatic metastasis of OCs (67, 68). According to the riskscore formula, the value of calculated riskscore showed positive correlation with WTAP expression level, which was consistent with previous study. Furthermore, the value of riskscore was also positively correlated with ZC3H13, whereas it was negatively correlated with ALYREF and METTL1. Although no specific clinical researches explore the association between OC prognosis and expression levels of ZC3H13, ALYREF, and METTL1, this present study emphasized their important roles in OC pathological features and prognosis.

Differentially enriched pathways were found between high-risk and low-risk groups. In high-risk group, significantly enriched pathways included calcium signaling pathway, focal adhesion, arrhythmogenic right ventricular cardiomyopathy (arvc), complement and coagulation cascades, vascular smooth muscle contraction, dilated cardiomyopathy, hypertrophic cardiomyopathy (hcm), and neuroactive ligand receptor interaction. Among them, focal adhesion was an important signaling pathway in cell migration (109), and calcium signaling pathway controlled multiple cell processes, such as cell proliferation and metabolism (110), which were consistent with features of OC cells. Other pathways like arrhythmogenic right ventricular cardiomyopathy (arvc), dilated cardiomyopathy, complement and coagulation cascades, vascular smooth muscle contraction, and hypertrophic cardiomyopathy (hcm), were all key pathways in disease of cardiovascular system, which indicated that drugs targeting these signaling pathways for cardiovascular diseases might have potential roles in reduction of OC risk and treatment. The discovery of differential enriched pathways provided ones with novel medication regimens to lower the risk of OCs through blocking these signaling pathways.

### Relationship between RNA methylation and identified differential immune cells/immune checkpoints/TMB and role in OCs

This present study found that the prognostic model-based riskscore was positively correlated with CD4+ memory resting T cells, and negatively correlated with plasma cells and macrophages M1, which demonstrated that high-risk group was dominated by high-level infiltration of CD4+ memory resting T cells. Both high-level of CD4+ memory resting T cells infiltration and low-level of plasma cells and macrophages M1 infiltration might imply poor prognosis of OC patients. CD4+ memory resting T cells were differentiated from naïve CD4+ T cells experiencing an antigen so that molecular alterations inevitably emerged in CD4+ memory resting T cells after exposure. A multi-omic comparative analysis showed that methylation levels of promoter regions in kinases LYN, SGK1, and transmethylase METTL7A, and hydrolase DDAH2 were elevated, and concurrently gene expression levels decreased (111). Plasma cells as antibody-secreting cells had tremendous speed of immunoglobulin-coding genes in transcription, translation, assembly and secretion. Plasma cells were differentiated from B cells with the help of IRF4 and Blimp-1. Blimp-1 and XBP1 were critical upstream regulatory factors of the unfolded protein response in plasma cells (112). Macrophages M1 were induced by IFN-γ with the function of intense bactericidal and anti-inflammatory effects. m6A writer METTL3 actuated macrophages M1 polarization through methylating STAT1 mRNA (113).

Immune checkpoint therapy was a novel and attention-getting tumor treatment, which could strengthen anti-tumor immune response of T cells with broad application prospects. CD276, also known as B7-H3, was a member of B7 family. A review summarized the role of CD276 in cancers, regulation mechanism and its potential therapeutic value (114). CD276 took part in the regulation of cell cycle, cell differentiation, proliferation, invasion, apoptosis, and epithelial-mesenchymal transition, and also participated in tumor metastasis. Moreover, in aspect of immune regulation, CD276 had synergistic effects with CTLA4, PD-1, PD-L1, and PD-L2 in inhibition of T cells proliferation and activation, and IFN-γ, TNF-α, and other cytokines secretion (114). This present study found that CD276 was a differential immune checkpoint molecule between high- and low-risk groups, which indicated its crucial function in OC progression and suggested that CD276 immune checkpoint inhibitors might have a considerable effect on OC immune therapy.

Tumor mutation burden (TMB) was an emerging potential biomarker for immune checkpoint blockade selection in diverse cancers. Mutation-derived neoantigens in tumor DNA could be identified and targeted by human immune system. After transcription and translation, peptides containing mutation-derived neoantigens could be processed and transferred to MHC molecules, and appear on the surface of cells. It is certain that the more mutations a tumor had, the more neoantigens it formed, and the more likely immune treatments would work (115). TMB has become an important predictor of immune checkpoint blockade outcomes and an available biomarker to identify patients who would benefit from immune therapy (115). A study found that high TMB was significantly correlated with better PFS and OS in OCs (116). This present study found that TMB was positively correlated with macrophages M1, T cells gamma delta, B cells memory, and negatively correlated with NK cells activated, and B cells naïve, which demonstrated that TMB could be estimated *via* immune cell infiltration and further contributed to immune therapy strategies of OCs. Although the experimental validation of LASSO model in clinical samples is able to strengthen a computational study, it is generally not required for a computational study; the use of extra database to validate it is also acceptable. Also, it is so difficult to collect enough samples to verify the LASSO model. Thus, we used extra database to verify our LASSO model, which provides us the preliminary work for the deep validation in real clinical samples in future.

## Conclusion

RNA modification and its regulation played a crucial role in tumorigenesis, progression, and prognosis of OC patients. The constructed four-DERRG signature (ALYREF, ZC3H13, WTAP, and METTL1) model might be an independent prognostic model to divide OC patients into high- and low-risk groups, which was of great significance for prognostic assessment, patient stratification, and predictive evaluation of immunotherapy outcomes in OCs.

## Data availability statement

The datasets presented in this study can be found in online repositories. The names of the repository/repositories and accession number(s) can be found in the article/**Supplementary Material**.

## Ethics statement

Written informed consent was not obtained from the individual(s) for the publication of any potentially identifiable images or data included in this article.

## Author contributions

PZ analyzed data and wrote the manuscript draft. NL conceived the concept, analyzed data, and wrote the manuscript. XZ conceived the concept, coordinated, critically revised manuscript, and was responsible for the corresponding works. All authors contributed to the article and approved the submitted version.

## Funding

This work was supported by the Shandong Cancer Hospital Qihang Plan (to NL), the Shandong First Medical University Talent Introduction Funds (to XZ), Shandong First Medical University High-level Scientific Research Achievement Cultivation Funding Program (to XZ), the Shandong Provincial Natural Science Foundation (ZR2021MH156 to XZ; and ZR2022QH112 to NL), Shandong Provincial Taishan Scholar Engineering Project Special Funds (to XZ), National Nature Scientific Funds (82203592 to NL; and the Academic Promotion Program of Shandong First Medical University (2019ZL002).

## Conflict of interest

The authors declare that the research was conducted in the absence of any commercial or financial relationships that could be construed as a potential conflict of interest.

## Publisher’s note

All claims expressed in this article are solely those of the authors and do not necessarily represent those of their affiliated organizations, or those of the publisher, the editors and the reviewers. Any product that may be evaluated in this article, or claim that may be made by its manufacturer, is not guaranteed or endorsed by the publisher.

## Glossary

**Table:**

ac4C	N4-acetylcytidine
ADP	adenosine diphosphate
AUC	area under curve
BCL-2	B cell lymphoma-2
cAMP	cyclic adenosine monophosphate
CCNG2	cyclin G2
CR	complete response
CSF-1	colony stimulating factor-1
CTLA4	cytotoxic T lymphocyte-associated protein 4;
CXCL	chemokine C-X-C motif ligand
DDAH2	dimethylarginine dimethylaminohydrolase 2
DERRG	differently expressed RNAmodification regulatory gene
DKK1	dickkopf-1
DTP	Developmental Therapeutics Program
EIF3C	eukaryotic initiation factor 3c
EZH2	enhancer of zeste homolog 2
FXR1	fragile X autosomal homolog 1
GSEA	gene-set enrichment analysis
hCTR1	human copper transporter 1;
H3K27me3	trimethylation of lys-27 in histone 3
Ψ	pseudouridine
IFN	interferon
IL	interleukin
IRF4	interferon regulatory factor 4
LYN	lck/yesrelated protein tyrosine kinase
m1A	N1-methyladenosine
m3C	3-methylcytidine
m5C	5-methylcytosine
m6A	N6-methyladenosine;
m6Am	2-O-dimethyladenosine
m7G	N7-methyladenosine
MAD	median absolute deviation
MEX3A	Mex-3 RNA Binding Family Member A
MHC	major histocompatibility complex
NCI	National Cancer Institute
NF-kB	nuclear factor kappa beta
NK	natural killer cell
NMF	nonnegative matrix factorization
OC	ovarian cancer
OCT4	octamer-binding transcription factor 4
ORR	objective response rate
OS	overall survival
OTUB1	OUT domain-containing ubiquitin aldehyde-binding protein 1
PABPC1	poly A binding protein 1
PCA	principal component analysis
PD	progressive disease
PD-1	programmed cell death protein-1
PD-L1	programmed cell death ligand-1
PD-L2	programmed cell death ligand-2
PFS	progressionfree- survival
PKM2	pyruvate kinase M2
PKP3	plakophilin 3
PPI	protein–protein interaction
PR	partial response
ROC	receiver operating characteristic
RRG	RNA-modification regulatory gene
rRNA	ribosomal RNA
SD	stable disease
SFRP2	secreted frizzled-related protein 2
SGK1	serum and glucocorticoid-induced protein kinase 1
SHP-2	SH2 domaincontaining protein tyrosine phosphatase-2
STAT1	signal transducer and activator of transcription 1
TCGA	The Cancer Genome Atlas
TGF	transforming growth factor
Th1	T-helper 1
TIM	tumor immune microenvironment
TMB	tumor mutation burden
TNF	tumor necrosis factor
Tregs	T cells regulatory
tRNA	transfer RNA
TROAP	trophininassociated protein
WDR4	WD repeat domain 4
XBP1	X-box binding protein 1
